# The pathogenicity of swan derived H5N1 virus in birds and mammals and its gene analysis

**DOI:** 10.1186/s12985-014-0207-y

**Published:** 2014-11-29

**Authors:** Kairat Tabynov, Abylay Sansyzbay, Nurlan Sandybayev, Muratbay Mambetaliyev

**Affiliations:** The Research Institute for Biological Safety Problems, Zhambylskaya oblast, Kordayskiy rayon, Gvardeiskiy, 080409 Republic of Kazakhstan

**Keywords:** H5N1, Avian influenza virus, Pathogenicity, Birds, Mammals, Virulence determinants

## Abstract

**Background:**

Highly pathogenic avian influenza (HPAI) H5N1 viruses continue to circulate in poultry and can infect and cause mortality in birds and mammals; the genetic determinants of their increased virulence are largely unknown. The main purpose of this work was to determine the correlation between known molecular determinants of virulence in different avian influenza virus (AIV) genes and the results of experimental infection of birds and mammals with AIV strain A/swan/Mangistau/3/06 (H5N1; SW/3/06).

**Methods and results:**

We examined the virulence of SW/3/06 in four species of birds (chickens, ducks, turkeys, geese) and five species of mammals (mice, guinea pigs, cats, dogs, pigs), and identified the molecular determinants of virulence in 11 genes (HA, NA, PB1, PB1-F2, PB2, PA, NS1, NS2, M1, M2 and NP). SW/3/06 does not possess the prime virulence determinant of HPAIV – a polybasic HA cleavage site – and is highly pathogenic in chickens. SW/3/06 replicated efficiently in chickens, ducks, turkeys, mice and dogs, causing 100% mortality within 1.6–5.2 days. In addition, no mortalities were observed in geese, guinea pigs, cats and pigs. The HI assay demonstrated all not diseased animals infected with the SW/3/06 virus had undergone seroconversion by 14, 21 and 28 dpi. Eleven mutations in the seven genes were present in SW/3/06. These mutations may play a role in the pathogenicity of this strain in chickens, ducks, turkeys, mice and dogs. Together or separately, mutations 228S-103S-318I in HA may play a role in the efficient replication of SW/3/06 in mammals (mice, dogs, pigs).

**Conclusions:**

This study provides new information on the pathogenicity of the newly-isolated swan derived H5N1 virus in birds and mammals, and explored the role of molecular determinants of virulence in different genes; such studies may help to identify key virulence or adaptation markers that can be used for global surveillance of viruses threatening to emerge into the human population.

**Electronic supplementary material:**

The online version of this article (doi:10.1186/s12985-014-0207-y) contains supplementary material, which is available to authorized users.

## Background

Influenza A viruses have eight-segmented, negative, single-stranded RNA genomes and are serologically divided into 18 hemagglutinin (HA, H1-H18) and eleven neuraminidase (NA, N1-N11) subtypes [1,2]. All known influenza A virus subtypes has been isolated from a number of species including humans, wild and domesticated birds, pigs, horses and sea mammals [3], and there is evidence of transmission to domestic dogs and cats [4]. The genome of influenza A viruses consists of eight unique segments of single-stranded, negative, sense RNA. The viral RNA segments encode ten recognized gene products: the polymerases PB1, PB2 and PA, and the proteins HA, NP, NA, M1, M2, NS1 and NS2 [3,5].

Viruses typically acquire critical alterations in their genomes that allow them to adapt to or severely damage their host. Identification of these genetic changes will improve our understanding of the determinants of virulence and aid in the development of counter measures against viral infection and spread [6]. A number of genetic determinants are associated with the virulence and ability of avian influenza viruses (AIVs) to replicate in mammalian cells [7]. Examination of the molecular mechanisms responsible for the adaptation of AIVs from natural reservoirs to new hosts is important for understanding the evolution of influenza viruses. The switch in host cell receptor specificity from sialic acid connected to galactose via α2-3 linkages (avian) to α2-6 linkages (mammalian) is a major obstacle that prevents AIVs from crossing the species barrier and adapting to new hosts [8].

Three influenza viral proteins, PB2, HA and NS1, are recognized as major determinants of virulence, pathogenicity and host range restriction [9,10]. HA cleavage is critical for virulence, as it exposes the hydrophobic N-terminus of HA2, which mediates fusion of the viral and endosomal membranes. The HA proteins of all highly pathogenic avian influenza (HPAI) H5N1 viruses contain multiple basic amino acids at their cleavage site [5]. The NS1 protein plays an important role in countering host cell antiviral cytokines and the initial host immune response in chickens and cattle [11,12]. Recently, it was shown that the amino acids at position 627 [13,14] and 701 [15,16] of the polymerase subunit PB2 and 97, 349 and 550 of the polymerase subunit PA [17,18] may play important roles in the adaptation of HPAI H5N1 viruses from birds to mammals.

Several studies have shown that mouse-adapted influenza A and B viruses possess unique modifications in a short stretch of the C-terminal domain of the M1 protein [19-21]. The M1 protein contributes to the virulence of HPAI H5N1 viruses in mammalian hosts; the amino acids Asp at position 30 and Ala at position 215 are necessary for the lethality of H5N1 viruses in mice [22]. In addition to the polybasic HA cleavage site, the caspase cleavage motif of the M2 protein and deletions within the stalk region of NA are associated with increased virulence of HPAI H5N1 viruses [23-25].

Continuing outbreaks of HPAI H5N1 world-wide emphasize the importance of full genetic characterization of different viral strains from a variety of countries, as well as pathogenicity studies in a range of animal species. The present study examined the correlation between known and novel molecular determinants of virulence in influenza genes with the results of experimental infection of birds (chickens, ducks, geese, turkeys) and mammals (mice, guinea pigs, cats, dogs, pigs) with the AIV strain A/swan/Mangistau/3/06 (H5N1; SW/3/06) that was isolated in 2006 from a dead swan found in the Mangistau region of the Republic of Kazakhstan. Identification of genetic determinants of virulence will help to provide to a better understanding of the mechanisms involved in influenza virus transmission and assist in pandemic preparedness.

## Results

### Epidemiology

In March 2006, mass wild bird mortalities were recorded on the coast of the Caspian Sea. As part of the investigation into this outbreak, staff from the Research Institute for Biological Safety Problems (RIBSP) selected 12 samples of pathological material (trachea, lung, intestine, brain) from three dead mute swans found on Cape Peschannyi, 70 km from the city of Aktau in the Mangistau region of Kazakhstan [26].

Analysis demonstrated that AIV A/H5N1 was the cause of death in the mute swans. The viral strain SW/3/06 was isolated from 10-day-old specific pathogen-free (SPF) embryonated chicken eggs (ECEs); molecular markers of pathogenicity associated with HPAI H5N1 were not present in the HA and NA surface proteins of this strain (the HA proteolytic cleavage site contains an amino acid sequence characteristic of low pathogenicity influenza viral strains) [26]. Nevertheless, the intravenous pathogenicity index (IVPI) of strain SW/3/06 in chickens was 2.34, indicating that the strain is a highly pathogenic [27]. This generated further interest in the study of the virulence of strain SW/3/06 in different species of birds and mammals, along with discovery of its molecular determinants of virulence.

### Genetic and phylogenetic analysis

The genotype of the *HA* gene of strain SW/3/06 (clade EA-nonGsGD) differs from that of strains previously isolated in Kazakhstan: A/domestic goose/Pavlodar/1/05 (H5N1; GS/1/05) and A/chicken/Astana/6/05 (H5N1; CK/6/05) related to clade 2.2 of the Qinghai genotype [28]. Strain SW/3/06 demonstrated antigenic shift; the virus did not react with the antisera of the strains isolated in Kazakhstan in 2005 [29], suggesting SW/3/06 is probably a reassortant strain of AIVs circulating in the Russian Far East and Japan as the highest degree of HA amino acid sequence homology was identified with strains A/duck/Hokkaido/vac-1/04 (H5N1) and A/duck/Primorie/2633/01 (H5N3), for which the percentage homology reached 96% [26,30].

This work established that the HPAI A/H5N1 viruses isolated in Kazakhstan have different NS genotypes. The strains isolated in 2005 (GS/1/05, CK/6/05) is referred to as the Qinghai genotype NS1E, whereas SW/3/06 has a NS2A genotype, which is typical of Gs/Gd-like strains [31]. On the basis of these results, Chervyakova et al. [31] suggested that strain SW/3/06 entered Kazakhstan from Europe (Sweden) via the Black Sea/Mediterranean migration routes of birds.

### Virus replication in experimentally-inoculated birds and mammals

#### Virulence and pathogenesis of A/swan/Mangistau/3/06 in different avian species

AIVs that kill 75% or more of eight intravenously (*i.v.*) inoculated chickens within 10 days are classified as highly pathogenic [32,33]. Inoculation of 4-week-old chickens with SW/3/06 virus led to 100% mortality (IVPI =2.34) within 3.3 days (Table 1).

**Table 1:** **Pathogenicity of the SW/3/06 virus in different avian species**

**Species (inoculation route)**	**Mortality** ^**a**^	**MDT (days)**	**Dose (EID**_**50**_ **)** ^**b**^	**Antibody titer on indicated day post-infection** ^**c**^		
				**14**	**21**	**28**
White Leghorns (*i.v*.)	10/10	3.3	10^4.3^	ND	ND	ND
White Leghorns (*i.n*.)	6/6	3.4	10^4.3^	ND	ND	ND
White Leghorns (*i.m*.)	6/6	3.6	10^4.3^	ND	ND	ND
Kholmogory geese (*i.n*.)	0/6	>28.0	10^4.3^	4.7 ± 0.6	6.0 ± 0.0	7.0 ± 1.0
Kholmogory geese (*i.m*.)	0/6	>28.0	10^4.3^	4.3 ± 0.6	5.3 ± 0.6	6.0 ± 0.0
Silver Bantam ducks (*i.n*.)	6/6	4.2	10^4.3^	ND	ND	ND
Silver Bantam ducks (*i.m*.)	6/6	5.2	10^4.3^	ND	ND	ND
Czech turkeys (*i.n*.)	6/6	1.6	10^4.3^	ND	ND	ND
Czech turkeys (*i.m*.)	6/6	1.8	10^4.3^	ND	ND	ND

^a^Number of birds that died/number of birds inoculated.

^b^Determined by back titer determination of the inoculum; EID_50_, 50% egg infectious dose.

^c^Presented as Log_2_ HI titer ± standard deviation. Antibody titers were determined by an HI assay of two-fold diluted serum.

ND, not determined.

Intranasal (*i.n.*) or intramuscular (*i.m*) inoculation of chickens, ducks and turkeys with 10^4.3^ EID_50_ of SW/3/06 virus also led to 100% mortality; however, the mean death times (MDTs) of chickens and ducks (3.4-3.6 and 4.2-5.2 days) were longer than that of *i.v.* inoculated chickens [27]. The MDT for *i.n.* and *i.m.* inoculated turkeys were shorter (1.6-1.8 days) than the other experimental groups of birds. In addition, no mortalities or clinical signs of disease were observed in geese *i.n.* or *i.m.* infected with SW/3/06, with seroconversion observed at 14, 21 and 28 days post-infection (dpi; Table 1).

**Figure 1:**
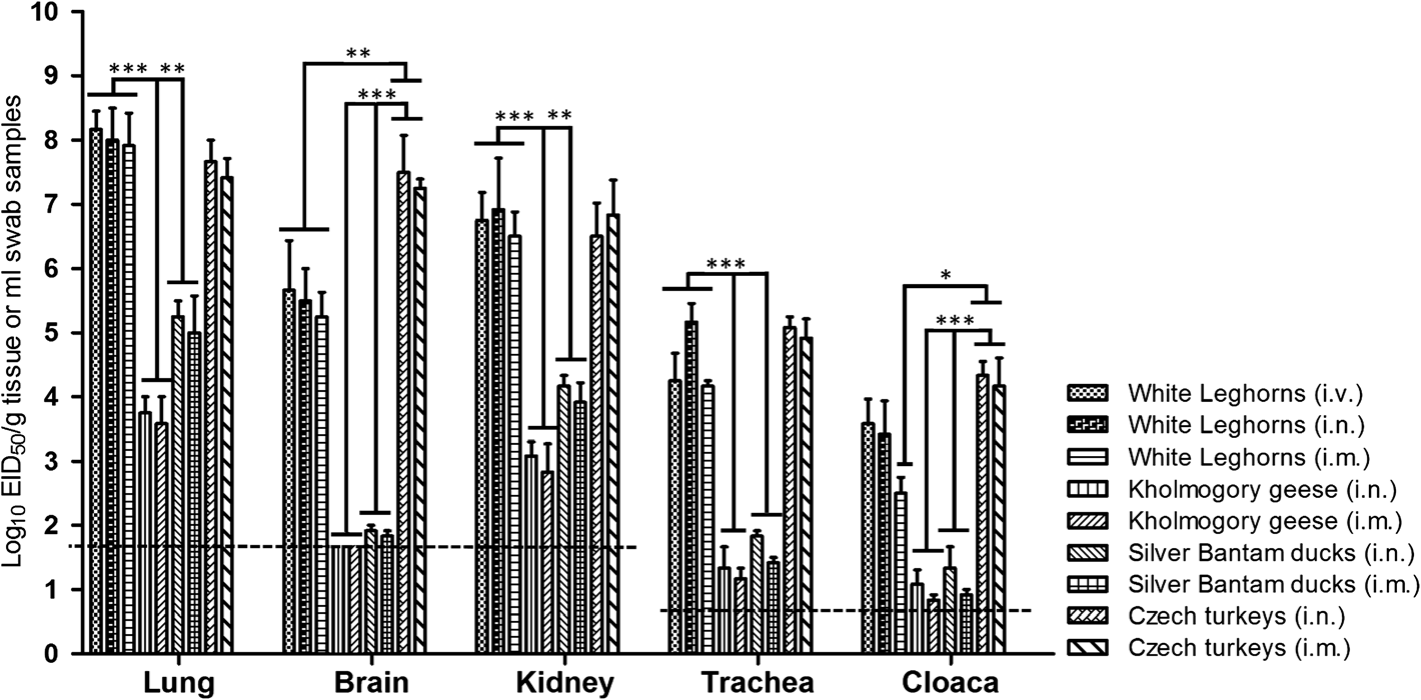
**Mean titers of SW/3/06 virus recovered from different avian species.** Chickens, ducks, turkeys and geese (six per group) were inoculated *i.n.* or *i.m*. with 10^4.3^ EID_50_/50 μl of SW/3/06 virus. Viral titers were determined in ECEs and expressed as EID_50_ per gram for tissue samples and EID_50_ per ml for tracheal and cloacal swabs. The limits of virus detection (horizontal dotted lines) for tissues and swabs were 1.75 and 0.75 log_10_ EID_50_, respectively (*P <0.05, **P <0.01, ***P <0.001; two-way ANOVA followed by Tukey's multiple comparisons test).

The SW/3/06 virus replicated and was shed from both the gastrointestinal and respiratory tracts of chickens, as indicated by the relatively high titers (3.6 to 5.2 log_10_ EID_50_/mL) of virus recovered from oropharyngeal and cloacal swabs (Figure 1). Furthermore, high AIV titers were also detected in kidney and brain tissues, as well as the lungs (5.7 to 8.2 log_10_ EID_50_/g of tissue). The tissue and swab viral titers were similar for both *i.v., i.n.* and *i.m*. inoculated chickens. SW/3/06 virus also grew rapidly in turkeys, as demonstrated by high viral titers in brain, kidney and lung tissues (6.8 to 7.7 log_10_ EID_50_/g) as well as oropharyngeal and cloacal swabs (4.3 to 5.0 log_10_ EID_50_/mL). Compared to chickens and turkeys, significantly lower virus accumulation was observed in the internal organs of the gastrointestinal and respiratory tracts of ducks and geese (from *P* <0.01 to *P* <0.001), and the SW/3/06 virus was not detected in the brains of any geese (< 10^1.75^ EID_50_/g; Figure 1). This study showed that geese shed the virus without exhibiting any clinical signs.

#### Virus replication and pathogenicity in mice

**Figure 2:**
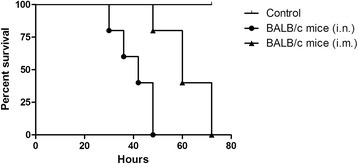
**Survival curves for mice inoculated with the SW/3/06 virus.** Female 5-week-old BALB/c mice (five per group) were inoculated *i.n.* or *i.m*. with 10^4.0^ EID_50_/50 μl of the SW/3/06 virus. Although they had equivalent mortality rates (100%), the *i.m.* inoculated group had significantly longer survival than the *i.n.* inoculated group (log-rank test, *P* =0.0005).

Five 5 to 6-week-old SPF female BALB/c mice (per group) were *i.n.* or *i.m.* inoculated with SW/3/06 virus under light sedation to assess virus replication and morbidity and mortality in mice. The infected mice displayed severe clinical signs, including ruffled fur, depression and labored breathing. All five mice in both groups died within 2.4-2.8 dpi (Table 2) with significantly longer survival observed in the *i.m.* inoculated group than the *i.n.* inoculated group (Figure 2; log-rank test, *P* =0.0005). Katz et al. [34] established that AIVs with a mouse lethal dose (MLD_50_) > 10^6.5^ were considered to have low pathogenicity, while MLD_50_ < 10^3.0^ were considered highly pathogenic in mouse models. The MLD_50_ of the SW/3/06 virus was < 10^3.0^ EID_50_ (Table 3); therefore, this virus is highly pathogenic in mice.

**Table 2:** **Pathogenicity of the SW/3/06 virus in different mammalian species**

**Species (inoculation route)**	**Mortality** ^**a**^	**MDT (days)**	**Dose (EID**_**50**_ **)** ^**b**^	**Antibody titer on indicated day post-infection** ^**c**^		
				**14**	**21**	**28**
BALB/c mice (*i.n.)*	5/5	2.4	10^4.0^	ND	ND	ND
BALB/c mice (*i.m*.)	5/5	2.8	10^4.0^	ND	ND	ND
Guinea pigs (*i.n*.)	0/5	>14.0	10^4.3^	5.0 ± 1.0	5.3 ± 0.6	5.7 ± 0.6
Guinea pigs (*i.m*.)	0/5	>14.0	10^4.3^	4.7 ± 1.1	4.7 ± 0.6	5.3 ± 0.6
Guinea pigs (*i.n*.)	0/3	>14.0	10^6.0^	5.0 ± 0.0	5.7 ± 0.6	6.0 ± 0.0
Guinea pigs (contact)	0/3	>14.0	–	< 1.0	< 1.0	< 1.0
Large White pigs (*i.n.*)	0/5	>14.0	10^5.3^	3.7 ± 0.6	4.0 ± 0.0	4.7 ± 0.6
Large White pigs (*i.m*.)	0/5	>14.0	10^5.3^	3.0 ± 0.0	3.3 ± 0.6	4.0 ± 0.0
Scottish Fold cats (*i.n*.)	0/3	>14.0	10^4.6^	7.0 ± 1.0	8.3 ± 1.1	8.7 ± 0.6
Scottish Fold cats (*i.t*.)	0/3	>14.0	10^4.6^	7.0 ± 1.7	8.0 ± 0.0	8.3 ± 0.6
Beagle dogs (*i.n*.)	3/3	2.3	10^4.6^	ND	ND	ND
Beagle dogs (*i.t*.)	3/3	4.7	10^4.6^	ND	ND	ND

^a^Number of mammals that died/number of mammals inoculated.

^b^Determined by back titer determination of the inoculum; EID_50_, 50% egg infectious dose.

^c^Presented as Log_2_ HI titer ± standard deviation. Antibody titers were determined by an HI assay of two-fold diluted serum.

ND, not determined.

**Table 3:** **Distribution of the SW/3/06 virus in experimentally inoculated mice**

**Inoculation route**	**Dose (EID**_**50**_ **)**	**Hpi**	**Viral titer (log**_**10**_ **EID**_**50**_ **/0.1 mL, mean ± standard deviation)**					**MLD**_**50**_ **(EID**_**50**_ **)**	**MID**_**50**_ **(EID**_**50**_ **)**
			**Lung**	**Brain**	**Liver**	**Spleen**	**Kidney**		
*I.n.*	10^4.0^	30-48	6.4 ± 0.7	1.9 ± 0.1	2.7 ± 0.6	4.2 ± 1.0	4.1 ± 0.8	2.9	1.7
*I.m.*	10^4.0^	48-72	6.2 ± 0.8	1.8 ± 0.1	2.0 ± 0.4	3.6 ± 0.5	3.5 ± 1.5		

BALB/c mice (five per group) were inoculated intranasally or intramuscularly with 10^4.0^ EID_50_/50 μl of SW/3/06 virus. To assess mouse LD_50_ (MLD_50_), the mice were inoculated with 10-fold serial dilutions of 10^6.0^ EID_50_ of SW/3/06 virus (10^0.0^ to 10^7.0^) via the intranasal route and observed for 14 days. To assess mouse ID_50_ (MID_50_), lung tissues were collected from three inoculated mice per group at 3 dpi. The lower virus detection limit was 10^1.75^ EID_50_/g.

Hpi, hour post infection.

To assess virus replication, we collected various organs such as lung, brain, liver, spleen and kidney from three dead mice in each group (inoculated with 10^4.0^ EID_50_ of SW/3/06 virus) and titrated virus replication in ECEs. The inoculated virus was recovered from all tested organs (mean viral titers, 10^1.8^ to 10^6.4^ EID_50_/0.1 mL) with no significant differences between the *i.n.* and *i.m.* inoculated groups (*P* =0.5130; Table 3). These results demonstrate that mice are highly susceptible to strain of SW/3/06 AIV.

#### Pathogenicity in dogs

**Figure 3:**
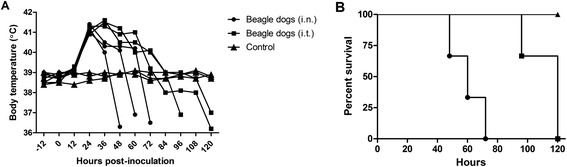
**Body temperature and survival curves of dogs inoculated with the SW/3/06 virus.** Eight-week-old specific pathogen-free Beagles (three per group) were inoculated *i.n*. or *i.t.* with 10^4.6^ EID_50_/mL of the SW/3/06 virus. **(A)** Body temperature after inoculation with SW/3/06 virus. **(B)** Survival curves. Although they had equivalent mortality rates (100%), the *i.t.* inoculated group had significantly longer survival than the *i.n.* inoculated group (log-rank test, *P* =0.0065).

The replication and virulence of the SW/3/06 virus were assessed in eight-week-old SPF beagles. Two groups of three dogs lightly anesthetized with ketamine were *i.n.* or intratracheally (*i.t.)* inoculated with 1 mL of 10^4.6^ EID_50_ of SW/3/06 virus. Nasal and rectal swabs were collected from the infected dogs on day 2 post-infection for viral titration in ECEs. Organs were collected from three dead animals in each group for assessment of virus replication and pathological analysis. All six dogs became anorexic on day 1 post-infection and completely lost their appetites. All animals developed fever around 24 h post-infection, and their temperature declined between 48 and 72 h (*i.n*.) or 96 and 120 h (*i.t.*) post-infection (Figure 3A). Conjunctivitis, diarrhea, cough and labored breathing were observed in all three animals in both groups. All animals died within 2.3-4.7 dpi (Table 2) with significantly longer survival observed in the *i.t.* inoculated group than the *i.n.* inoculated group (Figure 3B; log-rank test, *P* =0.0065).

**Figure 4:**
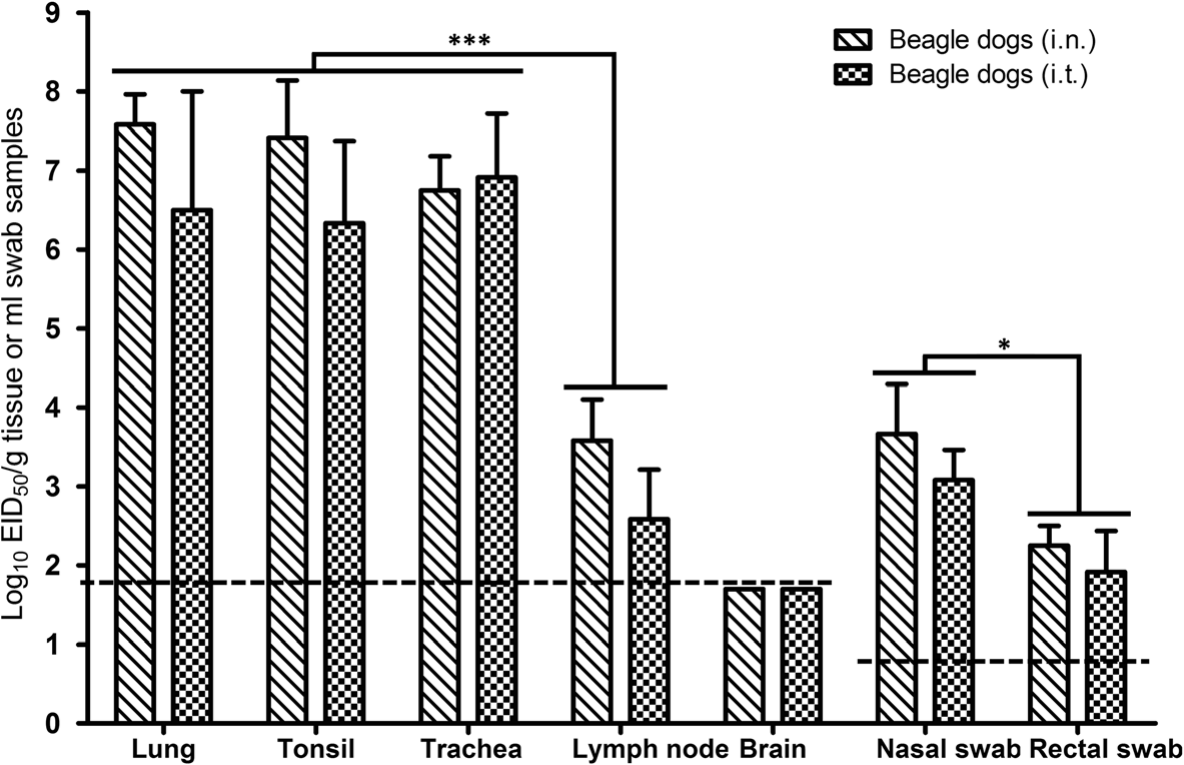
**Virus replication in beagles after inoculation with the H5N1 influenza virus strain SW/3/06.** Tissue homogenates were prepared from the animals that died post-infection with the SW/3/06 virus via either the *i.n*. or *i.t* route*.* Viral titers were determined in ECEs and expressed as EID_50_ per gram for tissue samples and EID_50_ per ml for tracheal and cloacal swabs. The limits of virus detection (horizontal dotted lines) for tissues and swabs were 1.75 and 0.75 log_10_ EID_50_, respectively (*P <0.05, ***P <0.001; two-way ANOVA followed by Tukey’s multiple comparisons test).

To investigate whether the inoculated dogs shed the virus, swabs titrated in ECEs. As shown in Figure 4, the virus was detected from the nasal and rectal swabs of all three animals in the *i.n.* and *i.t.* inoculated groups. The viral titers of the swabs were significantly higher in the *i.n.* group than *i.t.* group (*P* <0.05; Figure 4). In both groups, high viral titers were observed in the lung, tonsil and trachea, and low titers in the lymph nodes (*P* <0.001; Figure 4); the virus was not detected in the brain. These results indicate the SW/3/06 virus can replicate efficiently in dogs inoculated *i.n.* or *i.t.*

**Figure 5:**
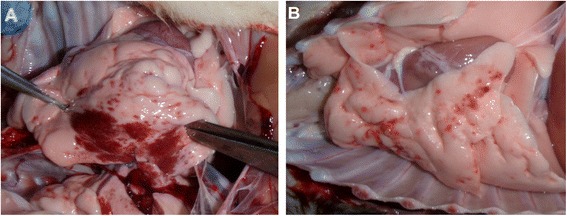
**Macroscopic lung lesions in beagles infected with the SW/3/06 virus. (A)** Macroscopic lung lesion in a beagle infected *i.n* with the virus on day 3 post-infection. Animals died 2.3 dpi. **(B)** Macroscopic lung lesion in a beagle infected *i.t.* with the virus on day 5 post-infection. Animals died 4.7 dpi.

Macroscopic lung lesions were detected in all six dogs. Multifocal hemorrhages were observed in the lungs of all dogs inoculated *i.n.* (Figure 5A) and *i.t.* (Figure 5B). The area of multifocal hemorrhage was more pronounced in the lungs of *i.n.* infected dogs than *i.t.* infected dogs. Prominent swelling of extrapulmonary organs and hyperemia of the tonsils were observed in all dogs. These results demonstrate that dogs are highly susceptible to strain of SW/3/06 AIV.

#### Pathogenicity in cats

Groups of 8–12-week-old cats were inoculated *i.n*. (*n* =5) or *i.t.* (*n* =5) with 10^4.6^ EID_50_ of the SW/3/06 virus. Five other cats were mock-infected with PBS as controls. No clinical abnormalities were observed in any group; the body temperature of cats inoculated *i.n.* or *i.t.* remained within physiological norms (data not shown). Virus was not isolated from any tissues or swabs (data not shown). However, cats inoculated *i.n.* or *i.t.* with the SW/3/06 virus had seroconverted at 14, 21, 28 dpi, and the HI assay demonstrated the serum contained high levels of antibodies against subtype H5 AIVs (Table 2). These results indicate that cats are not susceptible to experimental infection with a strain of SW/3/06 AIV.

#### Pathogenicity in guinea pigs

To investigate replication of the SW/3/06 virus in guinea pigs, ten animals were inoculated *i.n.* (*n* =5) or *i.m.* (*n* =5) with 10^4.3^ EID_50_ of SW/3/06 virus. Two animals from each group were euthanized 3 dpi; nasal washes and the trachea, lung, brain, kidney, spleen and colon were collected from each animal for viral titration in ECEs. The virus was detected in the nasal washes, tracheas and lungs of all animals inoculated *i.n*., but not in the nasal washes or tracheas of animal inoculated *i.m.* (Table 4). The virus was not detected in the brain, kidney, spleen or colon of any inoculated animal (data not shown). The remaining three animals in each group were observed for four weeks to detect signs of disease. At 2, 3 and 4 weeks post-infection, all animals had undergone seroconversion, as indicated by the HI assay (Table 2). None of the animals showed signs of disease during the period of observation. These results indicate that replication of the SW/3/06 virus is restricted to the respiratory system in guinea pigs.

**Table 4:** **Replication of the SW/3/06 virus in guinea pigs**

**Inoculation route (dose, EID**_**50**_ **)**	**Mean viral titer (log**_**10**_ **EID**_**50**_ **/g)** ^**a**^			**Seroconversion (positive/total)** ^**c**^
	**Nasal wash** ^**b**^	**Trachea**	**Lung**	
*I.n.* (10^4.3^)	2.2 ± 0.3	1.5 ± 0.7	3.6 ± 0.9	3/3
*I.m.* (10^4.3^)	–	–	1.8 ± 0.2	3/3

^a^Groups of five guinea pigs were slightly anesthetized and inoculated intranasally or intramuscularly with 10^4.3^ EID_50_ of test virus (300 μl volume, 150 μl per nostril). Virus was not detected in the spleen, kidney, colon or brain of any animal inoculated with the SW/3/06 virus; data not shown. –, virus not detected in undiluted samples.

^b^Log_10_ EID_50_/ml.

^c^Seroconversion was determined on 14, 21, 28 dpi by the HI assay.

**Figure 6:**
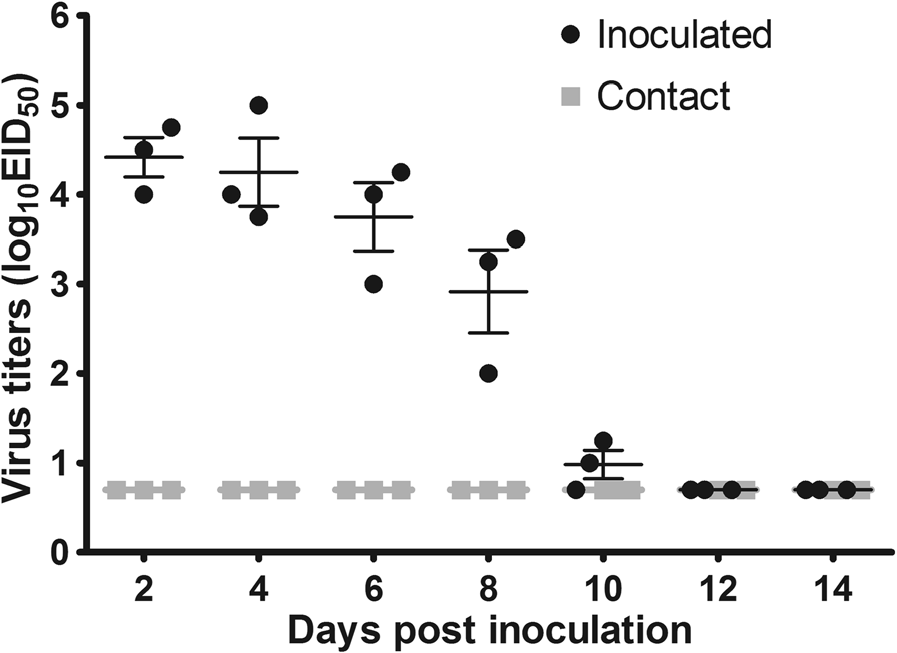
**Transmission of the SW/3/06 virus in guinea pigs.** Three guinea pigs were inoculated *i.n.* with 10^6^ EID_50_ of the SW/3/06 virus and, 24 h post-inoculation, three contact guinea pigs were placed in each cage. Nasal washes were collected every two days from all animals beginning 2 dpi for detection of virus shedding. Numbers of guinea pigs shedding virus are shown in each point and square. The lower virus limit detection was 10^0.75^ EID_50_/mL.

For the transmission study, three animals were inoculated *i.n.* with 10^6^ EID_50_ of the SW/3/06 virus and three naïve animals were introduced into the same cage 24 h post-infection Evidence of transmission was based on detection of virus in nasal washes and on seroconversion at the end of the four-week observation period. The virus was detected in the nasal washes of all inoculated guinea pigs between days 2–10 post-infection, but not in any of the contact guinea pigs (Figure 6).

Seroconversion occurred in all inoculated animals; however, seroconversion was not observed in the contact group (Table 2). These results indicate that the SW/3/06 virus does not transmit efficiently in this mammalian host.

#### Pathogenicity in pigs

Groups of 12-week-old piglets were *i.n.* (*n* =5) or *i.m.* (*n* =5) inoculated with 10^5.3^ EID_50_ of the SW/3/06 virus. Five other pigs were mock-infected with PBS as controls. No changes in food consumption or behavior were observed in any inoculated animal. The body temperature of the pigs inoculated with the SW/3/06 virus and the control animals remained with physiological norms (data not shown).

**Figure 7:**
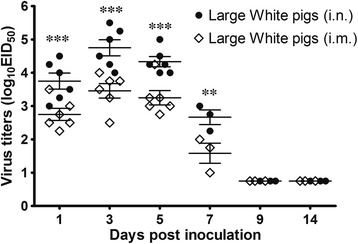
**Viral titers of nasal swabs collected from pigs inoculated with the SW/3/06 virus.** Each data point and rhombus represents the mean ± SEM viral titer (log_10_ EID_50_/ml of sample media) for pigs positive for the influenza virus in Rapid Test Kit Н5 AIV Ag. Numbers of pigs shedding virus are shown in each point and rhombus. The lower virus detection limit was 10^0.75^ EID_50_/mL (**P <0.01, ***P <0.001; two-way ANOVA followed by Tukey's multiple comparisons test).

To detect the virus and determine infective titers, nasal and rectal swabs were collected from the infected pigs. The virus was not detected in any rectal swabs. However, the virus was detected in nasal swabs taken on 1, 3, 5 and 7 dpi in all inoculated pigs (Figure 7). The viral titers of the swabs taken from the animals in the *i.n.* inoculated group were significantly higher than that of the *i.m*. inoculated group (from *P* <0.01 to *P* <0.001; Figure 7). In general, the H5N1 viral titers were similar in nasal samples collected from the *i.n.* and *i.m.* inoculated groups on days 1, 3 and 5, and were higher than the titer of samples collected on day 7 (*P* <0.01, Figure 7).

**Figure 8:**
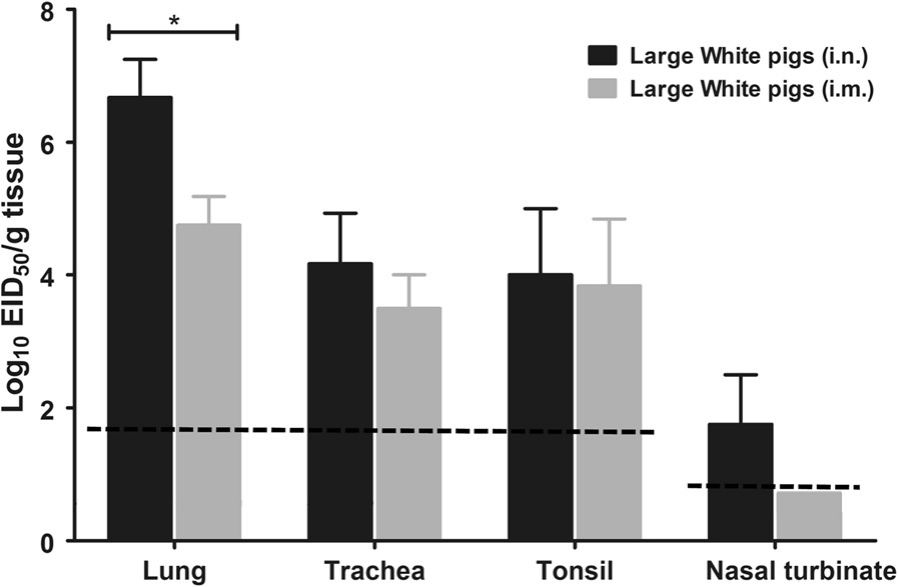
**Viral titers of tissues collected from pigs inoculated with the SW/3/06 virus.** Each bar represents the mean and range viral titer (log_10_ EID_50_/gram of tissue) for tissues collected from three pigs euthanized on day 5 post-inoculation. The limits of virus detection (horizontal dotted lines) for tissues and swabs were 1.75 and 0.75 log_10_ EID_50_, respectively (*P <0.05; two-way ANOVA followed by Tukey’s multiple comparisons test).

To determine the sites of virus replication, organ and tissue samples (lungs, trachea, tonsils, nasal turbinate, brain, heart, spleen, liver, kidney, adrenal glands) were collected from the infected pigs on day 5 post-infection. Virus was only detected in the respiratory organs of the inoculated pigs (Figure 8), namely the lungs, trachea, tonsils and nasal turbinate; high viral titers were detected in the lung, trachea and tonsils. The lung viral titers were significantly higher in the *i.n.* inoculated group than the *i.m.* inoculated group (*P* <0.05; Figure 8). Low virus accumulation was observed in the nasal turbinate of pigs *i.n.* inoculated; however, the virus was not detected in the nasal turbinate of pigs inoculated *i.m.* (< 10^0.75^ EID_50_/g; Figure 8). The HI assay demonstrated all pigs infected with the SW/3/06 virus had undergone seroconversion by 14, 21 and 28 dpi (Table 2). These results indicate that replication of the SW/3/06 virus is restricted to the respiratory system in pigs.

### Molecular determinants of virulence

Known molecular determinants of virulence for HPAI H5N1 viruses (Additional file 1: Table S1) were identified in the genes encoding HA (N140S or D140S), PB1 (L473V), PA (K237E, N383D), NS1 (D97E, V149A, C-terminal ESEV motif), M1 (N30D, T215A), M2 (cleavage VDVD↓DG89) and NP (A184K) in the strain SW/3/06. Other known molecular determinants of virulence were not identified, including proteolytic cleavage site at positions 339–346 of HA (H5 numbering), the 20-amino-acid deletion (positions 49–68) in the stalk region of NA, the E627K or D701N mutations of PB2, the P42S mutation of NS1, the M105V mutation of NP and the other mutations shown in Additional file 1: Table S1. However, these molecular determinants of virulence were identified in the GS/1/05 and CK/6/05 virus strains that were isolated in Kazakhstan (Additional file 1: Table S1).

The Kazakhstan strains SW/3/06, CK/6/05 and GS/1/05 all contain a glutamine residue at position 226 (H3 numbering) of HA, which has been associated with preferential binding of sialic acid on the host cells joined to sugar chains via an α-2,3 linkage; this mutation is typical of HPAI H5N1 viruses (Additional file 2: Table S2). However, the SW/3/06 strain also contained the mutations 228S and 103S in the HA gene, which are absent in strains CK/6/05 and GS/1/05. The 318I mutation of HA was also present in all tested strains (Additional file 2: Table S2).

## Discussion

Outbreaks of HPAI were recorded in four regions of Kazakhstan (Pavlodar, Akmola, Karaganda, North Kazakhstan) between July 22 and August 17, 2005. Four strains of HPAI A/H5N1 viruses (GS/1/05, A/chicken/Karaganda/4/05, A/chicken/NKO/5/05, CK/6/05) were isolated from the epizootic nidus; these strains are related to clade 2.2 of the Qinghai genotype [28,35-38]. The IVPI of these strains in chickens ranges from 2.59-2.69, and the strains cause 100% mortality in chickens within 2–4 days of infection [27]. A second wave of outbreaks of HPAI was registered on the coast of the Caspian Sea in March 2006. The strain SW/3/06 (H5N1) was subsequently isolated from autopsy materials collected from dead mute swans found on Cape Peschannyi, 70 km from the city of Aktau in the Mangistau region of Kazakhstan [26].

In this study, we examined a virus with a wild-bird origin, SW/3/06 (clade EA-nonGsGD), that does not possess the prime virulence determinant of HPAI H5N1 viruses: a polybasic HA cleavage site [Additional file 1: Table S1]. However, the SW/3/06 virus is highly pathogenic in chickens (IVPI =2.34) [27]; therefore, this strain is a highly pathogenic. Therefore, we found it necessary to examine the virulence of strain SW/3/06 in four species of birds (chickens, ducks, turkeys, geese) and five species of mammals (mice, guinea pigs, cats, dogs, pigs), and furthermore, identify the molecular determinants of virulence in the11 genes of this strain (HA, NA, PB1, PB1-F2, PB2, PA, NS1, NS2, M1, M2 and NP).

Previous studies showed that H5N1 AIVs have varied pathogenic potential in avian and mammalian species, ranging from the complete absence of clinical disease to severe neurological dysfunction and death [39,40]. This study demonstrated that the SW/3/06 virus replicated readily in three bird species (chickens, ducks, turkeys) inoculated *i.n.* or *i.m.,* causing 100% mortality within 1.6–5.2 days. High titers of virus were shed from both the gastrointestinal and respiratory tracts of chickens and turkeys on day 3 post-infection; high titers were also detected in the lung, kidney and brain tissues. However, the viral titers of ducks and geese, in which a mild systemic infection was observed, were lower compared to those of chickens and turkeys. In addition, no mortalities were observed in geese infected *i.n.* or *i.m.,* and virus was not detected in the brain of any goose. Previous reports have suggested that domestic geese (*Anser domesticus*) played a key role in the evolution of Asian-lineage HPAI H5N1 viruses [41]. As geese vaccinated with inactivated H5N1 vaccines do not appear to develop prolonged, high-level immunity under experimental conditions without exposure to multiple, high doses of the vaccine [42], this species has the potential to play a role in viral persistence and spread if vaccination programs are irregularly employed, especially if lower doses than those recommended for chickens are used. This study showed that geese shed the virus without exhibiting any clinical signs, suggesting that geese may play an important role in the transmission of HPAI H5N1 viruses.

We also evaluated the susceptibility of dogs to H5N1 viral infection using beagles. The results demonstrate that dogs (beagles) are highly susceptible to the H5N1 influenza virus and may potentially serve as an intermediate host to transfer this virus to humans. Previous studies reported limited viral shedding and an inability to re-isolate virus from the organs of dogs experimentally inoculated with H5N1 viruses [43,44]. Songserm et al. [45] reported that an H5N1 influenza virus was detected in multiple organs of a dog that died from eating duck carcasses infected with H5N1 virus. However, in this study, efficient virus replication was detected in the respiratory system of all animals and viral shedding was detected in all *i.n.* and *i.t.* inoculated animals, in agreement with data obtained by Chen et al. in dogs infected *i.n.* [46]. The ability of H5N1 influenza viruses to replicate in dogs may vary from strain to strain, as observed in our previous study [47], in which dogs were not sensitive to experimental viral challenge with CK/6/05 (clade 2.2). This study demonstrates that the strain SW/3/06 can replicate much more efficiently in dogs than the strains tested by other researchers [42,43]. Therefore, effort should be made to prevent dogs from being infected by H5N1 influenza viruses to avoid the generation of a virus with pandemic potential in this host.

The virus strains circulating in Asia could possibly have a higher pathogenicity in cats than the strains of the European lineage [48]. Our results demonstrated that - in contrast to dogs -domesticated cats are not sensitive to infection with H5N1. It is possible that the insensitivity of cats to experimental infection with the SW/3/06 strain is associated with the origin of this strain, which presumably entered Kazakhstan from Europe (Sweden) via the Black Sea/Mediterranean migration routes of birds [49]. However, our previous studies [50] indicated that the strain A/chicken/NKO/5/05 (clade 2.2 of the Qinghai genotype) isolated in 2005 in Kazakhstan was highly pathogenic in cats; the clinical signs and results of pathological analysis of cats in our study of strain SW/3/06 (H5N1) were similar to those of an experimental study conducted in 2004 by Kuiken et al. [51].

Guinea pigs have been successfully employed as models to evaluate the transmissibility of AIV strains and other influenza viruses in mammalian hosts [52]. Our transmissibility study indicates that the SW/3/06 AIV strain does not transmit efficiently in this mammalian host. Replication of the SW/3/06 virus was restricted to the respiratory system in guinea pigs and none of the animals showed any signs of disease. However, the presence of the virus in the nasal washes, trachea and lungs of all animals inoculated *i.n.* suggests that the guinea pig is worthy of further exploration as an animal model for studying the nonlethal respiratory infections induced by some HPAI H5N1 viruses.

This study indicated that HPAI H5N1 viruses can replicate in Large White piglets; however, these animals had a low susceptibility to infection. Infected Large White pigs shed higher viral titers than Landrace-Large White cross piglets inoculated with H5N1 viruses (clade 1, clade 2.1, clade 2.2 and clade 2.3), and the time of shedding (7 dpi) and viral titers of the lungs, trachea and tonsils were similar to that of Landrace-Large White cross piglets infected with H1N1 and H3N2 swine influenza viruses, as reported by Lipatov et al. [53]. Additionally, seroconversion occurred in all *i.n.* and *i.m.* infected pigs. These results indicate that H5N1 AIVs can be transmitted to pigs, which may increase the risk of these viral genes being transmitted into humans.

The SW/3/06 virus replicated efficiently in chickens, ducks, turkeys, mice and dogs. However, the amino acid mutations in the protein PB2, NS1, NP, HA, and NA that are known to contribute to the pathogenicity of HPAI H5N1 viruses in birds and mammals ([23], Additional file 1: Table S1) were not observed in this strain. However, we identified 11 mutations in seven genes (HA, N140S or D140S); PB1 (L473V); PA (K237E, N383D); NS1 (D97E, V149A, C-terminal ESEV motif); M1 (N30D, T215A); M2 (cleavage VDVD↓DG89); NP (A184K)) in the strain SW/3/06 ([6,11,20,22,24], Additional file 1: Table S1); these mutations are likely to play a role in the pathogenicity of the SW/3/06 strain in chickens, ducks, turkeys, mice and dogs.

The HA gene of the SW/3/06 strain also contains the 228S mutation, which has been associated with switching from SA-α-2,3-Gal to SA-α-2,6-Gal host receptor specificity ([54], Additional file 2: Table S2), as well as the 103S mutation; these mutations are absent in the strains CK/6/05 and GS/1/05. The HA 318I mutation, which is associated with reduced binding to avian-type receptors and increased binding to human-type receptors, was also in present in all strains tested [Additional file 2: Table S2]. It is possible that - either together or separately - the 228S-103S-318I mutations in the HA gene play an important role in the efficient replication of the SW/3/06 strain in mammals (mice, dogs, pigs).

In summary, we have described the pathogenicity of the newly-isolated HPAI H5N1 viral strain SW/3/06 in birds and mammals, and explored the role of molecular determinants of virulence in different genes. Additional research to obtain mutant strains using reverse genetics is required in order to fully characterize the role of the mutations identified in the genes of strain SW/3/06; these studies may help to identify key virulence or adaptation markers that can be used for global surveillance of viruses threatening to emerge into the human population. In addition, such research may provide insights into new opportunities to combat such cross-species transfer and potentially prevent a pandemic that could emerge at the human-animal interface.

## Materials and methods

### Facility

Studies of HPAI H5N1 viruses were conducted in a biosecurity level 3 laboratory approved by the Ministry of Health of the Republic of Kazakhstan. Animal experiments were conducted according to protocols approved by the Institutional Animal Care and Use Committee based on applicable laws and guidelines (Permit Number: 07/10/177). Animals were housed in conventional animal facilities and received water and food ad libitum.

### Virus

The A/swan/Mangistau/3/06 (SW/3/06) virus was isolated from the pathological materials (trachea, lung, intestine, brain) of a dead mute swan found in the Mangistau region, Kazakhstan [26]. The virus was propagated in 10-day-old specific-pathogen-free (SPF) embryonated chicken eggs (ECEs) for 60 h at 35°C and then stored at −70°C.

### Animal experiments

#### Intravenous pathogenicity index test

The intravenous pathogenicity index (IVPI) of the AIV isolates was determined as described in the OIE Manual of Standards for Diagnostic Tests and Vaccines [32].

#### Pathogenicity studies in birds

An intranasal (*i.n*.) and intramuscular (*i.m*.) inoculation study was conducted to determine the pathogenicity of the virus in four types of birds. Twelve 6-week-old White Leghorn chickens (*G. gallus domesticus*), twelve 6-week-old Kholmogory geese (*Anser anser*), twelve 6-week-old Silver Bantam ducks (*Anas platyrhyncos*) and 12 four-week-old Czech turkeys (*Meleagris gallopavo*) were inoculated *i.n.* or *i.m.* with 10^4.3^ EID_50_ of the SW/3/06 virus. All birds were observed daily for 4 weeks. Tracheal and cloacal swabs were collected on day 3 post-infection and examined for the presence of influenza virus using Rapid Test Kit Н5 AIV Ag (Animal Genetics, Inc., Suwon-si, Korea). Sera were collected at 2 and 3 weeks post-infection and at the end of the experiment (4 weeks post-infection).

Three clinically normal birds from each group of geese were euthanized on day 3 post-infection by administration of sodium pentobarbital (100 mg/kg body weight *i.v.*). In addition, all chicken, ducks and turkeys that died were pathologically evaluated for gross lesions and tissue samples (lung, brain, kidney) were collected for virus isolation. Tissues were weighed and homogenized in sterile phosphate-buffered saline (PBS), and the infectivity of the clarified homogenates was titrated in embryonated chicken eggs (ECEs). For swab samples, the swab medium was clarified before inoculation into ECEs.

#### Mouse inoculation experiments

Female, 5 to 6-week-old BALB/c mice (Charles River Laboratories, Sulzfeld, Germany; five per group) were inoculated *i.n.* or *i.m.* with 10^4.0^ EID_50_ of virus in a volume of 0.05 mL under light anesthesia induced using Zoletil (Virbac S.A., La Seyne-sur-Mer, France). The viral titers in the kidney, liver, spleen, lung and brain were examined in three dead mice from each group. The tissues were collected, homogenized in 1 mL PBS, centrifuged, the supernatant was diluted 1:10 and virus replication was titrated in ECEs. To determine the mean mouse lethal dose (MLD_50_) and the 50% mouse infectious dose (MID_50_), eight 6-week-old mice per group were inoculated *i.n.* with 0.0 to 10^7.0^ EID_50_/50 μl of 10-fold serially-diluted SW/3/0610 virus. For MID_50_, lung tissues were sampled from three mice euthanized days 2 post-inoculation per group, and virus replication in the tissue homogenates was determined in ECEs. The remaining five mice per group were observed for 14 days to determine the MLD_50_; MLD_50_ was calculated using the method of Reed & Muench [55].

#### Dog inoculation experiments

Eight to twelve-week-old Beagle dogs (*n* =9; Biotest S.r.o., Konárovice, Czech Republic) were serologically tested using the HI assay with 0.5% chicken red blood cells (CRBCs); all dogs were seronegative for H5 influenza viruses. The dogs were infected *i.n.* (n =3) or intratracheally (*i.t*.; *n* =3) with 10^4.6^ EID_50_ of SW/3/06 virus. Three other dogs were mock-infected with PBS as controls. Rectal body temperature was assessed every day for 5 dpi. Swabs were collected from the trachea and rectum on day 2 post-infection, suspended in PBS, and used to inoculate 10-day-old ECEs to determine the log_10_ EID_50_/mL. Trachea, lymph nodes, tonsil, brain and lung tissues were collected from the dead dogs; the tissues of mock-infected (control) dogs euthanized on day 5 post-infection were also collected. Tissues were weighed, ground in sterile PBS containing antibiotics to prepare 10% homogenates and injected into 10 day-old ECEs for viral titration.

#### Cat inoculation experiments

Eight to twelve-week-old Scottish Fold cats (*n* =15; Scottish Fold Cattery, Bellissimo, Republic of Kazakhstan) were serologically tested using the HI assay with 0.5% CRBCs; all animals were seronegative for H5 influenza viruses. The cats were infected *i.n.* (*n* =5) or *i.t.* (*n* =5) with 10^4.6^ EID_50_ of the SW/3/06 virus. Five other cats were mock-infected with PBS as controls. Rectal body temperature was measured for 14 dpi. Swabs were collected from the trachea and rectum every day, suspended in PBS, and used to inoculate White Leghorn 10-day-old ECEs to determine the log_10_ EID_50_/mL. Clinical signs were observed for 28 days after infection. Two cats from each group were euthanized on day 5 post-infection via intravenous injection of T61 (0.3 mL/kg body weight; Intervet Ln, Millsboro, USA); lung, brain, spleen, kidney, liver tissues were collected, weighed, ground in sterile PBS containing antibiotics to prepare 10% homogenates, and injected into 10 day-old ECEs for viral titration. The remaining animals were observed and serum samples were collected on 14, 21 and 28 dpi and analyzed using the HI assay with 0.5% CRBCs according to standard procedures [33].

#### Guinea pig inoculation experiments

Female Hartley strain guinea pigs (Charles River Laboratories, Wilmington, MA, USA) weighing 300–350 g that were seronegative for influenza virus were used. Ketamine (20 mg/kg) and xylazine (1 mg/kg) were used to anesthetize the animals via *i.n.* or *i.m.* White Leghorn *i.n.* or *i.m.* with 10^4.3^ EID_50_ of the SW/3/06 virus (volume, 300 μl; 150 μl per nostril). Three animals from each group were euthanized on day 3 post-infection and nasal washes, trachea, lung, brain, kidney, spleen and colon tissue samples were collected for viral titration in ECEs. The remaining two animals were observed for four weeks for signs of disease and serum samples were collected on 14, 21, 28 dpi for the HI assay using 0.5% CRBCs according to standard procedures [33]. For contact transmission studies, groups of five animals were inoculated *i.n.* with 10^6^ EID_50_ of the SW/3/06 virus and housed in cages placed inside an isolator. Five naïve animals were introduced into the same cages 24 h later. Nasal washes were collected at 2 day intervals, beginning on day 2 post-infection (1 day post-contact) and titrated in ECEs. To prevent inadvertent physical transmission of virus by the investigators, the contact guinea pigs were always handled first, and gloves, implements and napkins on the work surface were changed between animals. The ambient conditions for these studies were 20–22°C and 30–40% relative humidity. The airflow in the isolator was horizontal with a speed of 0.1 m/s.

#### Pig inoculation experiments

Twelve-week-old male castrated pigs (Large White) were purchased from a local commercial farm. The pigs had not received any vaccines on the production farm. In the animal laboratory facilities, the pigs were housed in High Efficiency Particulate Air (HEPA) filtered isolation units and allowed to acclimatize for seven days. Piglets were fed commercially-available pelleted diet in amounts prescribed by the manufacturer to fulfill all dietary needs. Each virus treatment group consisted of six pigs that were anesthetized via *i.m.* injection of a ketamine (20 mg/kg) and xylazine (2 mg/kg) mixture and inoculated *i.n.* or *i.m.* with a dose 10^5.3^ EID_50_ of virus. Six other pigs were mock-infected with sterile PBS as controls. Body temperature and feed consumption were monitored daily, starting 1 day before inoculation and ending on day 14 post-infection.

Nasal and rectal swabs were collected 3 days before infection and on days 1, 3, 5, 7, 9 and 14 post-infection. Swabs were tested in 10 day-old ECEs to detect and titrate the virus (lower detection limit, 10^0.75^ EID_50_/mL). Before titration, each sample of allantoic fluid that was positive in a hemagglutination test was confirmed to be influenza A/H5 virus positive using the Anigen Rapid Test Kit Н5 AIV Ag (Animal Genetics, Inc., Suwon-si, Korea). Viral titers were expressed as log_10_ EID_50_ per 1 mL of swab media. Three pigs from each group were euthanatized on day 5 post-infection, and lung, trachea, tonsil, nasal turbinate, brain, heart, spleen, liver, kidney, adrenal gland samples were collected, weighed, ground in sterile PBS containing antibiotics to prepare 10% homogenates, and injected into 10 day-old ECEs for virus detection and titration.

Pigs were bled one day before and on day 14, 21 and 28 post-infection. To destroy non-specific inhibitors, the serum samples were heat-inactivated at 56°C for 30 min and then treated with 10% CRBCs for 60 min at 4°C. Serum antibody titers were determined using the hemagglutination inhibition (HI) assay with 0.5% CRBCs according to standard procedures [33].

### Sequencing of complete genomes

Viral RNA was extracted from allantoic fluid using the RNeasy mini kit (Qiagen, Valencia, CA, USA). RT-PCR was performed to amplify gene segments of the SW/3/06 virus using a set of previously described universal primers [56]. The PCR products were purified by agarose gel electrophoresis and the Qiagen gel purification kit (Qiagen). The nucleotide sequences of the HA (Genbank Accession No.: FJ436942), NA (FJ436943), NS (JF262041), PA, PB1, PB2, NP and MP (not yet published in GenBank) genes were determined by dideoxy sequencing using an ABI Genetic Analyser 3130xl (Applied Biosystems, Foster, CA, USA).

### Statistical analyses

Data were analyzed using Prism v.5.01 (GraphPad Software, Inc., San Diego, CA, USA), and values are expressed as the mean ± standard error of the mean. Kaplan-Meier survival rates were compared using the log rank test; two-way ANOVA followed by Tukey’s multiple comparisons test was used to analyze virus infectivity in the tissues and swabs of infected birds and mammals. Statistical significance was defined as *P* <0.05.

## Additional files

Additional file 1: Table S1. Amino acid mutations involved in H5N1 viral infection and pathogenicity in birds and mammals. Additional file 2: Table S2. HA mutations associated with H5N1 influenza viruses switching from avian to human receptor specificity.

## References

[1] Tong S, Zhu X, Li Y, Shi M, Zhang J, Bourgeois M, Yang H, Chen X, Recuenco S, Gomez J, Chen LM, Johnson A, Tao Y, Dreyfus C, Yu W, McBride R, Carney PJ, Gilbert AT, Chang J, Guo Z, Davis CT, Paulson JC, Stevens J, Rupprecht CE, Holmes EC, Wilson IA, Donis RO (2013). New world bats harbor diverse influenza A viruses. PLoS Pathog.

[2] Fouchier RA, Munster V, Wallensten A, Bestebroer TM, Herfst S, Smith D, Rimmelzwaan GF, Olsen B, Osterhaus AD (2005). Characterization of a novel influenza A virus hemagglutinin subtype (H16) obtained from black-headed gulls. J Virol.

[3] Webster RG, Bean WJ, Gorman OT, Chambers TM, Kawaoka Y (1992). Evolution and ecology of influenza A viruses. Microbiol Rev.

[4] Amonsin A, Songserm T, Chutinimitkul S, Jam-on R, Sae-Heng N, Pariyothorn N, Payungporn S, Theamboonlers A, Poovorawan Y (2007). Genetic analysis of influenza A virus (H5N1) derived from domestic cat and dog in Thailand. Arch Virol.

[5] Neumann G, Chen H, Gao GF, Shu Y, Kawaoka Y (2010). H5N1 influenza viruses: outbreaks and biological properties. Cell Res.

[6] Song J, Feng H, Xu J, Zhao D, Shi J, Li Y, Deng G, Jiang Y, Li X, Zhu P, Guan Y, Bu Z, Kawaoka Y, Chen H (2011). The PA protein directly contributes to the virulence of H5N1 avian influenza viruses in domestic ducks. J Virol.

[7] Zhang H, Hale BG, Xu K, Sun B (2013). Viral and host factors required for avian H5N1 influenza A virus replication in mammalian cells. Viruses.

[8] Parrish CR, Kawaoka Y (2005). The origins of new pandemic viruses: the acquisition of new host ranges by canine parvovirus and influenza A viruses. Annu Rev Microbiol.

[9] Neumann G, Kawaoka Y (2006). Host range restriction and pathogenicity in the context of influenza pandemic. Emerg Infect Dis.

[10] Neumann G, Shinya K, Kawaoka Y (2007). Molecular pathogenesis of H5N1 influenza virus infections. Antivir Ther.

[11] Li Z, Jiang Y, Jiao P, Wang A, Zhao F, Tian G, Wang X, Yu K, Bu Z, Chen H (2006). The NS1 gene contributes to the virulence of H5N1 avian influenza viruses. J Virol.

[12] Tabynov K, Kydyrbayev Z, Ryskeldinova S, Yespembetov B, Zinina N, Assanzhanova N, Kozhamkulov Y, Inkarbekov D, Gotskina T, Sansyzbay A (2014). Novel influenza virus vectors expressing Brucella L7/L12 or Omp16 proteins in cattle induced a strong T-cell immune response, as well as high protectiveness against B.abortus infection. Vaccine.

[13] Govorkova EA, Rehg JE, Krauss S, Yen HL, Guan Y, Peiris M, Nguyen TD, Hanh TH, Puthavathana P, Long HT, Buranathai C, Lim W, Webster RG, Hoffmann E (2005). Lethality to ferrets of H5N1 influenza viruses isolated from humans and poultry in 2004. J Virol.

[14] Hatta M, Gao P, Halfmann P, Kawaoka Y (2001). Molecular basis for high virulence of Hong Kong H5N1 influenza A viruses. Science.

[15] Li Z, Chen H, Jiao P, Deng G, Tian G, Li Y, Hoffmann E, Webster RG, Matsuoka Y, Yu K (2005). Molecular basis of replication of duck H5N1 influenza viruses in a mammalian mouse model. J Virol.

[16] Steel JA, Lowen C, Mubareka S, Palese P (2009). Transmission of influenza virus in a mammalian host is increased by PB2 amino acids 627 K or 627E/701 N. PLoS Pathog.

[17] Rolling T, Koerner I, Zimmermann P, Holz K, Haller O, Staeheli P, Kochs G (2009). Adaptive mutations resulting in enhanced polymerase activity contribute to high virulence of influenza A virus in mice. J Virol.

[18] Song MS, Pascua PN, Lee JH, Baek YH, Lee OJ, Kim CJ, Kim H, Webby RJ, Webster RG, Choi YK (2009). The polymerase acidic protein gene of influenza a virus contributes to pathogenicity in a mouse model. J Virol.

[19] Brown EG, Liu H, Kit LC, Baird S, Nesrallah M (2001). Pattern of mutation in the genome of influenza A virus on adaptation to increased virulence in the mouse lung: identification of functional themes. Proc Natl Acad Sci U S A.

[20] Govorkova EA, Gambaryan AS, Claas EC, Smirnov YA (2000). Amino acid changes in the hemagglutinin and matrix proteins of influenza a (H2) viruses adapted to mice. Acta Virol.

[21] Lee KH, Youn JW, Kim HJ, Seong BL (2001). Identification and characterization of mutations in the high growth vaccine strain of influenza virus. Arch Virol.

[22] Fan S, Deng G, Song J, Tian G, Suo Y, Jiang Y, Guan Y, Bu Z, Kawaoka Y, Chen H (2009). Two amino acid residues in the matrix protein M1 contribute to the virulence difference of H5N1 avian influenza viruses in mice. Virology.

[23] Baigent SJ, McCauley JW (2001). Glycosylation of haemagglutinin and stalk-length of neuraminidase combine to regulate the growth of avian influenza viruses in tissue culture. Virus Res.

[24] Zhirnov OP, Klenk HD (2009). Alterations in caspase cleavage motifs of NP and M2 proteins attenuate virulence of a highly pathogenic avian influenza virus. Virology.

[25] Munier S, Larcher T, Cormier-Aline F, Soubieux D, Su B, Guigand L, Labrosse B, Cherel Y, Quéré P, Marc D, Naffakh N (2010). A genetically engineered waterfowl influenza virus with a deletion in the stalk of the neuraminidase has increased virulence for chickens. J Virol.

[26] Sandybayev NT, Strochkov VM, Sultankulova KT, Zholdybayeva YV, Zaitsev VL, Mamadaliyev SM (2008). Sequencing And Genetic Analysis Of Hemagglutinin And Neuraminidase Of A/mute swan/Mangystau/3/06 Strain Of Avian Influenza (H5N1) Virus. Proceedings of the International Conference on Biotechnology in Kazakhstan: Problems And Prospects Of Innovative Progress: 19–21 May 2008; Almaty.

[27] Tabynov KK, Mambetaliyev MA, Mamadaliyev MS, Bulatov YA, Kopochenya AA (2008). Assessment Of The Intravenous Pathogenicity Index Of Avian Influenza Virus Strains Isolated On The Territory of Kazakhstan in 2005–2006. Proceedings of the International Conference on Biotechnology in Kazakhstan: problems and prospects of innovative progress: 19–21 May 2008; Almaty.

[28] Sandybayev NT, Strochkov VM, Mamadaliyev SM, Zaitsev VL, Sultankulova KT, Chervyakova OV, Mambetaliyev M, Kydyrbayev ZK, Tabynov KK (2010). Genetic And Phylogenetic Analysis Of Avian Influenza A/H5N1 Virus Strains Isolated In Kazakhstan. Proceedings of the Sixth International Conference on Bioresources and viruses: 14–17 September 2010; Kiev.

[29] Tabynov KK, Mamadaliyev MS, Azanbekova MA, Mambetaliyev M (2009). Antigenic relationship of highly pathogenic avian influenza A/H5N1 viruses isolated on the territory of the Republic of Kazakhstan in 2005–2006. Actual Issues Vet Biol.

[30] Sandybayev NT, Strochkov VM, Mamadaliyev SM, Zaitsev VL, Sultankulova KT, Chervyakova OV, Mambetaliyev M, Kydyrbayev ZK, Tabynov KK (2010). Phylogenetic Analysis Of High - And Low Pathogenic Strains Of Avian Influenza Virus A/H5N1 Isolated in Kazakhstan in 2005–2007. Proceedings of the International Conference on Modern State of Genetics in Kazakhstan: 9 November 2010; Almaty.

[31] Chervyakova OV, Strochkov VM, Sultankulova KT, Sandybayev NT, Zaitsev VL, Mamadaliyev SM (2011). Molecular and genetic analysis of NS gene from high pathogenic strains of the avian influenza (H5N1) virus isolated in Kazakhstan. Gene.

[32] Office International des Epizooties, World organization for Animal Health (2001). “Highly pathogenic avian influenza (fowl plague),” in Manual of Standards for Diagnostic Tests and Vaccines.

[33] WHO (2002). WHO Manual On Animal Influenza Diagnosis And Surveillance.

[34] Katz JM, Lu X, Tumpey TM, Smith CB, Shaw MW, Subbarao K (2000). Molecular correlates of influenza A H5N1 virus pathogenesis in mice. J Virol.

[35] Mamadaliyev SM, Koshemetov ZK, Matveyeva VM, Kydyrbayev ZK, Zaitsev VL, Khairullin BM, Mambetaliyev MA, Sandybayev NT, Nurabayev SS, Azhibayev AZ, Bulatov YA, Katubayeva BS, Kozhamkulov YM, Tabynov KK, Kydyrmanov AI, Daulbayeva KD, Shahvorostova LI (2007). Avian influenza virus H5N1 subtype A diagnosed in sick and dead wild and domestic birds in Pavlodar oblast, Republic of Kazakhstan. Afr J Agric Res.

[36] Sansyzbay AR, Erofeeva MK, Khairullin BM, Sandybayev NT, Kydyrbayev ZK, Mamadaliyev SM, Kassenov MM, Sergeeva MV, Romanova JR, Krivitskaya VZ, Kiselev OI, Stukova MA (2013). An inactivated, adjuvanted whole virion clade 2.2 H5N1 (A/chicken/Astana/6/05) influenza vaccine is safe and immunogenic in a single dose in humans. Clin Vaccine Immunol.

[37] Tabynov KK, Sansyzbai AR, Biyashev BK, Mambetaliyev M (2013). Isolation and identification of highly pathogenic avian influenza virus A/H5N1 in Kazakhstan. J Sci Shakarim’ Semey State Univ.

[38] Tabynov KK (2014). Isolation and identification of highly pathogenic avian influenza A/H5N1 in Karaganda, North-Kazakhstan and Mangistau regions of Kazakhstan. Vet Sci.

[39] Isoda N, Sakoda Y, Kishida N, Bai GR, Matsuda K, Umemura T, Kida H (2006). Pathogenicity of a highly pathogenic avian influenza virus, A/chicken/Yamaguchi/7/04 (H5N1) in different species of birds and mammals. Arch Virol.

[40] Perkins LE, Swayne DE (2003). Comparative susceptibility of selected avian and mammalian species to a Hong Kong-origin H5N1 high-pathogenicity avian influenza virus. Avian Dis.

[41] FAO: Available at http://www.fao.org/avianflu/documents/key_ai/key_book_ch3.PDF.

[42] Tian G, Zhang S, Li Y, Bu Z, Liu P, Zhou J, Li C, Shi J, Yu K, Chen H (2005). Protective efficacy in chickens, geese and ducks of an H5N1-inactivated vaccine developed by reverse genetics. Virology.

[43] Giese M, Harder TC, Teifke JP, Klopfleisch R, Breithaupt A, Mettenleiter TC, Vahlenkamp TW (2008). Experimental infection and natural contact exposure of dogs with avian influenza virus (H5N1). Emerg Infect Dis.

[44] Maas R, Tacken M, Ruuls L, Koch G, van Rooij E, Stockhofe-Zurwieden N (2007). Avian influenza (H5N1) susceptibility and receptors in dogs. Emerg Infect Dis.

[45] Songserm T, Amonsin A, Jam-on R, Sae-Heng N, Pariyothorn N, Payungporn S, Theamboonlers A, Chutinimitkul S, Thanawongnuwech R, Poovorawan Y (2006). Fatal avian influenza A H5N1 in a dog. Emerg Infec Dis.

[46] Chen Y, Zhong G, Wang G, Deng G, Li Y, Shi J, Zhang Z, Guan Y, Jiang Y, Bu Z, Kawaoka Y, Chen H (2010). Dogs are highly susceptible to H5N1 avian influenza virus. Virology.

[47] Tabynov KK, Sansyzbay AR, Biyashev BK, Mambetaliyev M (2013). Spectrum pathogenicity of avian influenza virus strain A/chicken/Astana/6/05 (H5N1) for birds and different types of animals. J Sci Shakarim’ SSu.

[48] Salzberg SL, Kingsford C, Cattoli G, Spiro DJ, Janies DA, Aly MM, Brown IH, Couacy-Hymann E, De Mia GM, Dung do H, Guercio A, Joannis T, Maken Ali AS, Osmani A, Padalino I, Saad MD, Savić V, Sengamalay NA, Yingst S, Zaborsky J, Zorman-Rojs O, Ghedin E, Capua I (2007). Genome analysis linking recent European and African influenza (H5N1) viruses. Emerg Infec Dis.

[49] Xu X, Subbarao K, Cox NJ, Guo Y (1997). Genetic characterization of the pathogenic influenza A/Goose/Guangdong/1/96 (H5N1) virus: similarity of its hemagglutinin gene to those of H5N1 viruses from the 1997 outbreaks in Hong Kong. Virology.

[50] Tabynov KK, Nurgaziev RZ, Sansyzbai AR, Mambetaliyev M (2014). Pathogenicity in birds and mammals of avian influenza virus A/H5N1 isolated in North Kazakhstan region. Online J HAC KR.

[51] Kuiken T, Rimmelzwaan G, van Riel D, van Amerongen G, Baars M, Fouchier R, Osterhaus A (2004). Avian H5N1 influenza in cats. Science.

[52] Lowen AC, Mubareka S, Tumpey TM, Garcia-Sastre A, Palese P (2006). The guinea pig as a transmission model for human influenza viruses. Proc Natl Acad Sci U S A.

[53] Lipatov AS, Kwon YK, Sarmento LV, Lager KM, Spackman E, Suarez DL, Swayne DE (2008). Domestic pigs have low susceptibility to H5N1 highly pathogenic avian influenza viruses. PLoS Pathog.

[54] Vines A, Wells K, Matrosovich M, Castrucci MR, Ito T, Kawaoka Y (1998). The role of influenza A virus hemagglutinin residues 226 and 228 in receptor specificity and host range restriction. J Virol.

[55] Reed LJ, Muench H (1938). A simple method for estimating fifty percent endpoints. Am J Hyg.

[56] Hoffmann E, Stech J, Guan Y, Webster RG, Perez DR (2001). Universal primer set for the full-length amplification of all influenza A viruses. Arch Virol.

