# Supplementary material for: The pathogenicity of swan derived H5N1 virus in birds and mammals and its gene analysis
Source: Virol J. 2014 Nov 29;11:207. doi: 10.1186/s12985-014-0207-y (PMC4264262; doi:10.1186/s12985-014-0207-y)
Supplement: Additional file 1: Table S1. — Amino acid mutations involved in H5N1 viral infection and pathogenicity in birds and mammals. [file 12985_2014_207_MOESM1_ESM.docx]

Additional file 1: Table S1. Amino acid mutations involved in H5N1 viral infection and pathogenicity in birds and mammals

| Site | Residue | | Reference^b^ | Kazakhstan A/H5N1 strains | | |
| --- | --- | --- | --- | --- | --- | --- |
|  | Virulent | Non-virulent |  | SW/3/06 | CK/6/05 | GS/1/05 |
| HA polybasic cleavage  (RERRRKKR) | Yes | No | 1, 2, 3, 4, 5 | No | Yes | Yes |
| HA-102 | V | A, I, P, S, T | 6, 7 | D | I | I |
| HA-140 | S | N, D | 6, 7 | S | S | S |
| HA-154 | L, N | Q, H, I | 6, 7 | C | C | C |
| HA-172 | T, S | A | 6, 7, 8, 9 | K | K | K |
| HA-226 | L | Q | 1, 10, 11, 12 | Q | Q | Q |
| HA-228 | E, R | K | 1, 6, 7 | S | L | L |
| HA-279 | T | A | 6, 7 | G | G | G |
| NA stalk deletion (49:68 or 54:72) | Yes | No | 1, 13, 14 | No | Yes | Yes |
| PB1-207 | R | K | 15 | K | –^a^ | K |
| PB1-317 | I | M, V | 16, 7, 9, 50 | M | – | M |
| PB1-436 | Y | Y | 15 | Y | – | Y |
| PB1-473 | V | L | 18 | V | – | V |
| PB1-598 | P | L | 18 | L | – | K |
| PB1-F2-66 | S | N | 19 | N | – | – |
| PB2-318 | K | R | 7, 9 | R | – | – |
| PB2-355 | K | R, Q | 7, 9, 16, 17 | R | – | – |
| PB2-591 | K | Q | 19, 20 | Q | – | Q |
| PB2-627 | K | E | 1, 5, 9, 17, 21, 22 | E | – | K |
| PB2-701 | K, N | E, D | 1, 23, 24, 25 | D | – | – |
| PA-127 | I | V | 7, 26 | V | – | V |
| PA-224 | P | S | 27 | S | – | S |
| PA-336 | M | L | 7, 26 | L | – | L |
| PA-237 | E | K | 28 | E | – | E |
| PA-383 | D | N | 27 | D | – | D |
| PA-515 | A | T | 15 | T | – | T |
| NS1 (C-terminal ESEV motif) | Yes | No | 29, 30, 31, 32, 33 | Yes | No | No |
| NS1-42 | S | A, P | 7, 34 | A | S | S |
| NS1-92 | E | D | 1, 7, 17 | D | D | D |
| NS1-97 | E | D | 7, 35 | E | E | E |
| NS1-127 | N | T, D, R, V, A | 7, 36 | R | T | T |
| NS1-149 | A | V | 37 | A | A | A |
| NS1-189 | N | D, G | 7, 27 | D | D | D |
| NS1-195 | T, Y | S | 7, 38 | S | S | S |
| NS1-228 | P | S | 7, 30, 39 | S | S | S |
| NS2-31 | I | M | 7, 27 | M | M | M |
| NS2-56 | Y | H, L | 7, 27 | H | H | H |
| M1-30 | D | N | 40, 41 | D | D | D |
| M1-179 | K | M | 40 | M | M | M |
| M1-214 | H | Q | 40 | Q | Q | Q |
| M1-215 | A | T | 41 | A | A | A |
| M2 cleavage  (VDVD↓DG_89_) | Yes | No | 42 | Yes | No | – |
| M2-26 | I | L | 43 | L | L | – |
| M2-27 | A | V | 44 | V | V | – |
| M2-31 | N | S | 43 | S | S | – |
| M2-93 | S | N | 40 | N | – | – |
| NP cleavage  (METD↓G17) | Yes | No | 42 | No | No | No |
| NP-105 | V | M | 45 | M | V | V |
| NP-109 | T | I | 46 | I | I | I |
| NP-184 | K | A | 47 | K | K | K |

^a^Segment/protein not present in GenBank. The distribution of mutation identiﬁed in the literature were examined across strains. Abbreviations: HA, hemagglutinin; NA, neuraminidase; PB, polymerase basic; PA, polymerase acidic; NS, nonstructural protein; M, matrix protein; NP, nucleoprotein.

^b^Reference:

1. Tang Y, Wu P, Peng D, Wang X, Wan H, Zhang P, Long J, Zhang W, Li Y, Wang W, Zhang X, Liu X: Characterization of duck H5N1 influenza viruses with differing pathogenicity in mallard (Anas platyrhynchos) ducks. *Avian Pathol* 2009, 38(6):457-467.

2. Bosch FX, OrlichM, Klenk HD, Rott R: The structure of the hemagglutinin, a determinant for the pathogenicity of influenza viruses. *Virology* 1979, 95(1):197-207.

3. Horimoto T, Kawaoka Y: Reverse genetics provides direct evidence for a correlation of hemagglutinin cleavability and virulence of an avian influenza A virus. *J Virol* 1994, 68(5):3120-3128.

4. Senne DA, Panigrahy B, Kawaoka Y, Pearson JE, Suss J, Lipkind M, Kida H, Webster RG: Survey of the hemagglutinin (HA) cleavage site sequence of H5 and H7 avian influenza viruses: amino acid sequence at the HA cleavage site as a marker of pathogenicity potential. *Avian Dis* 1996, 40(2): 425-437.

5. Schat KA, Bingham J, Butler JM, Chen L-M, Lowther S, Crowley TM, Moore RJ, Donis RO, Lowenthal JW: Role of position 627 of PB2 and the multibasic cleavage site of the hemagglutinin in the virulence of H5N1 avian influenza virus in chickens and ducks. *PLoS One* 2012, 7(2):e30960.

6. Wu WL, Chen Y, Wang P, Song W, Lau SY, Rayner JM, Smith GJD, Webster RG, Peiris JSM, Lin T, Xia N, Guan Y, Chen H: Antigenic proﬁle of avian H5N1 viruses in Asia from 2002 to 2007. *J Virol* 2008, 82(4):1798-1807.

7. Lycett SJ, Ward MJ, Lewis FI, Poon AFY, Kosakovsky Pond SL, Leigh Brown AJ: Detection of mammalian virulence determinants in highly pathogenic avian influenza H5N1 viruses: multivariate analysis of published data. *J Virol* 2009, 83(19):9901-9910.

8. Bright RA, Ross TM, Subbarao K, Robinson HL, Katz JM: Impact of glycosylation on the immunogenicity of a DNA-based inﬂuenza H5 HA vaccine. *Virology* 2003, 308(2):270-278.

9. Chen H, Bright RA, Subbarao K, Smith C, Cox NJ, Katz JM, Matsuoka Y: Polygenic virulence factors involved in pathogenesis of 1997 Hong Kong H5N1 inﬂuenza viruses in mice. Virus Res 2007, 128(1-2):159-163.

10. Rogers GN, Paulson JC, Daniels RS, Skehel JJ, Wilson IA, Wiley DC: Single amino acid substitutions in influenza haemagglutinin change receptor binding specificity. *Nature* 1983, 304(5921):76-78.

11. Matrosovich M., Tuzikov A., Bovin N, Gambaryan A, Klimov A, Castrucci MR, Donatelli I, Kawaoka Y: Early alterations of the receptor-binding properties of H1, H2, and H3 avian influenza virus hemagglutinins after their introduction into mammals. *J Virol* 2000, 74(18):8502-8512.

12. Matrosovich MN, Matrosovich TY, Gray T, Roberts NA, Klenk HD: Human and avian influenza viruses target different cell types in cultures of human airway epithelium. *Proc Natl Acad Sci USA* 2004, 101(13):4620-4624.

13. Matrosovich M, Zhou N, Kawaoka Y, Webster R. The surface glycoproteins of H5 influenza viruses isolated from humans, chickens, and wild aquatic birds have distinguishable properties. *J Virol* 1999, 73(2):1146-1155.

14. Baigent SJ, McCauley JW: **Glycosylation of haemagglutinin and stalk-length of neuraminidase combine to regulate the growth of avian influenza viruses in tissue culture.** *Virus Res* 2001, **79**:177-185.

15. Hulse-Post DJ, Franks J, Boyd K, Salomon R, Hoffmann E, Yen HL, Webby RJ, Walker D, Nguyen TD, Webster RG: Molecular changes in the polymerase genes (PA and PB1) associated with high pathogenicity of H5N1 inﬂuenza virus in mallard ducks. *J Virol* 2007, 81(16):8515-8524.

16 Katz JM, Lu X, Tumpey TM, Smith CB, Shaw MW, Subbarao K: Molecular correlates of inﬂuenza A H5N1 virus pathogenesis in mice. *J Virol* 2000, 74(22):10807-10810.

17. Lee MS, Deng MC, Lin YJ, Chang CY, Shieh HK, Shiau JZ, Huang CC: Characterization of an H5N1 avian inﬂuenza virus from Taiwan. *Vet Microbiol* 2007, 124(3-4):193-201.

18. Xu C, Hu WB, Xu K, He YX, Wang TY, Chen Z, Li TX, Liu JH, Buchy P, Sun B: Amino acids 473V and 598P of PB1 from an avian-origin influenza A virus contribute to polymerase activity, especially in mammalian cells. *J Gen Virol* 2012, 93(3):531-540.

19. Conenello GM, Zamarin D, Perrone LA, Tumpey T, Palese P: A single mutation in the PB1-F2 of H5N1 (HK/97) and 1918 influenza A viruses contributes to increased virulence. *PLoS Pathog* 2007, 3(10):e141.

20. Yamada S, Hatta M, Staker BL, Watanabe S, Imai M, Shinya K, Sakai-Tagawa Y, Ito M, Ozawa M, Watanabe T, Sakabe S, Li C, Kim JH, Myler PJ, Phan I, Raymond A, Smith E, Stacy R, Nidom CA, Lank SM, Wiseman RW, Bimber BN, O'Connor DH, Neumann G, Stewart LJ, Kawaoka Y: Biological and structural characterization of a host-adapting amino acid in influenza virus. *PLoS Pathog* 2010, 6(8):e1001034.

21. Hatta M, Hatta Y, Kim JH, Watanabe S, Shinya K, Nguyen T, Lien PS, Le QM, Kawaoka Y: Growth of H5N1 influenza A viruses in the upper respiratory tracts of mice. *PLoS Pathog* 2007, 3(10):1374-1379.

22. Chen H, Deng G, Li Z, Tian G, Li Y, Jiao P, Zhang L, Liu Z, Webster RG, Yu K: The evolution of H5N1 inﬂuenza viruses in ducks in southern China. *Proc Natl Acad Sci USA* 2004, 101(28):10452-10457.

23. Steel JA, Lowen C, Mubareka S, Palese P: **Transmission of inﬂuenza virus in a mammalian host is increased by PB2 amino acids 627K or 627E/701N.** *PLoS Pathog* 2009, **5**:e1000252.

24. Le QM, Sakai-Tagawa Y, Ozawa M, Ito M, Kawaoka Y: Selection of H5N1 inﬂuenza virus PB2 during replication in humans. *J Virol* 2009, 83(10):5278-5281.

25. de Jong MD, Simmons CP, Thanh TT, Hien VM, Smith GJD, Chau TNB, Hoang DM, Van Vinh Chau N, Khanh TH, Dong VC, Qui PT, Van Cam B, Ha DQ, Guan Y, Peiris JSM, Chinh NT, Hien TT, Farrar J: Fatal outcome of human inﬂuenza A (H5N1) is associated with high viral load and hypercytokinemia. *Nat Med* 2006, 12(10):1203-1207.

26. Subbarao K, Shaw MW: Molecular aspects of avian inﬂuenza (H5N1) viruses isolated from humans. *Rev Med Virol* 2000, 10(5):337-348.

27. Song J, Feng H, Xu J, Zhao D, Shi J, Li Y, Deng G, Jiang Y, Li X, Zhu P, Guan Y, Bu Z, Kawaoka Y, Chen H: **The PA protein directly contributes to the virulence of H5N1 avian influenza viruses in domestic ducks.** *J Virol* 2011, **85**(5)**:**2180–2188.

28. Hu J, Hu Z, Mo Y, Wu Q, Cui Z, Duan Z, Huang J, Chen H, ChenY, Gu M, Wang X, Hu S, Liu H, Liu W, Liu X, Liu X: The PA and HA gene-mediated high viral load and intense innate immune response in the brain contribute to the high pathogenicity of H5N1 avian influenza virus in mallard ducks. *J Virol* 2013, 87(20):11063-11075.

29. Zielecki F, Semmler I, Kalthoff D, Voss D, Mauel S, Gruber AD, Beer M, Wolff T: Virulence determinants of avian H5N1 influenza A virus in mammalian and avian hosts: role of the C-terminal ESEV motif in the viral NS1 protein. J Virol 2010, 84(20):10708.

30. Jackson D, Hossain MJ, Hickman D, Perez DR, Lamb RA: A new influenza virus virulence determinant: the NS1 protein four C-terminal residues modulate pathogenicity. *Proc Natl Acad Sci USA* 2008, 105(11):4381-4386.

31. Golebiewski L, Liu H, Javier RT, Rice AP: The avian influenza virus NS1 ESEV PDZ binding motif associates with Dlg1 and Scribble to disrupt cellular tight junctions. *J Virol* 2011, 85(20):10639-10648.

32. Liu H, Golebiewski L, Dow EC, Krug RM, Javier RT, Rice AP: The ESEV PDZ-binding motif of the avian influenza A virus NS1 protein protects infected cells from apoptosis by directly targeting Scribble. *J Virol* 2010, 84(21):11164-11174.

33. Thomas M, Kranjec C, Nagasaka K, Matlashewski G, Banks L: Analysis of the PDZ binding specificities of Influenza A virus NS1 proteins. *Virol J* 2011, 8:25.

34. Jiao P, Tian G, Li Y, Deng G, Jiang Y, Liu C, Liu W, Bu Z, Kawaoka Y, Chen H: A single-amino-acid substitution in the NS1 protein changes the pathogenicity of H5N1 avian inﬂuenza viruses in mice. *J Virol* 2008, 82(3):1146-1154.

35. Long JX, Peng DX, Liu YL, Wu YT, Liu XF: Virulence of H5N1 avian inﬂuenza virus enhanced by a 15-nucleotide deletion in the viral nonstructural gene. *Virus Genes* 2008, 36(3):471-478.

36. Min JY, Li S, Sen GC, Krug RM: A site on the inﬂuenza A virus NS1 protein mediates both inhibition of PKR activation and temporal regulation of viral RNA synthesis. *Virology* 2007, 363(1):236-243.

37. Li Z, Jiang Y, Jiao P, Wang A, Zhao F, Tian G, Wang X, Yu K, Bu Z, Chen H: **The NS1 gene contributes to the virulence of H5N1 avian inﬂuenza viruses.** *J Virol* 2006, **80**:11115-11123.

38. Bornholdt ZA, Prasad BVV: X-ray structure of NS1 from a highly pathogenic H5N1 inﬂuenza virus. *Nature* 2008, 456(7224):985-988.

39. Obenauer JC, Denson J, Mehta PK, Su X, Mukatira S, Finkelstein DB, Xu X, Wang J, Ma J, Fan Y, Rakestraw K. M, Webster RG, Hoffmann E, Krauss S, Zheng J, Zhang Z, Naeve CW: Large-scale sequence analysis of avian inﬂuenza isolates. *Science* 2006, 311(5767):1576-1580.

40. Govorkova EA, Gambaryan AS, Claas EC, Smirnov YA: **Amino acid changes in the hemagglutinin and matrix proteins of inﬂuenza a (H2) viruses adapted to mice.** *Acta Virol* 2000, **44**(5)**:**241-248.

41. Fan Sh, Deng G, Song J, Tian G, SuoY, Jiang Y, Guan Y, Bu Z, Kawaoka Y, Chen H: **Two amino acid residues in the matrix protein M1 contribute to the virulence difference of H5N1 avian inﬂuenza viruses in mice.** *Virology* 2009, **384**(1)**:**28-32.

42. Zhirnov OP, Klenk HD: **Alterations in caspase cleavage motifs of NP and M2 proteins attenuate virulence of a highly pathogenic avian influenza virus.** *Virology* 2009, **394**(1)**:**57-63.

43. Govorkova EA, Baranovich T, Seiler P, Armstrong J, Burnham A, Guan Y, Peiris M, Webby RJ, Webster RG: Antiviral resistance among highly pathogenic influenza A (H5N1) viruses isolated worldwide in 2002-2012 shows need for continued monitoring. *Antiviral Res* 2013, 98(2):297-304.

44. Ilyushina NA, Govorkova EA, Webster RG: Detection of amantadine-resistant variants among avian influenza viruses isolated in North America and Asia. *Virology* 2005, 341(1):102-106.

45. Tada T, Suzuki K, Sakurai Y, Kubo M, Okada H, Itoh T, Tsukamoto K: NP body domain and PB2 contribute to increased virulence of H5N1 highly pathogenic avian influenza viruses in chickens. *J Virol* 2011, 85(4):1834-1846.

46. Tada T, Suzuki K, Sakurai Y, Kubo M, Okada H, Itoh T, Tsukamoto K: Emergence of avian influenza viruses with enhanced transcription activity by a single amino acid substitution in the nucleoprotein during replication in chicken brains. *J Virol* 2011, 85(19):10354-10363.

47. Wasilenko JL, Sarmento L, Pantin-Jackwood MJ: A single substitution in amino acid 184 of the NP protein alters the replication and pathogenicity of H5N1 avian influenza viruses in chickens. *Arch Virol* 2009, 154(6):969-79.
