# Supplementary material for: The pathogenicity of swan derived H5N1 virus in birds and mammals and its gene analysis
Source: Virol J. 2014 Nov 29;11:207. doi: 10.1186/s12985-014-0207-y (PMC4264262; doi:10.1186/s12985-014-0207-y)
Supplement: Additional file 2: Table S2. — HA mutations associated with H5N1 influenza viruses switching from avian to human receptor specificity. [file 12985_2014_207_MOESM2_ESM.docx]

Additional file 2: Table S2**.** HA mutations associated with H5N1 influenza viruses switching from avian to human receptor specificity

| Mutation | Reference^c^ | Kazakhstan A/H5N1 strains | | |
| --- | --- | --- | --- | --- |
|  |  | SW/3/06 | CK/6/05 | GS/1/05 |
| E119G^a^ | 1, 2 | E | E | E |
| V152I^a^ | 1, 2 | S | S | S |
| N158D^a^ | 1, 2 | G | G | G |
| N224K^a^ | 1, 2 | L | L | L |
| Q226L^a^ | 1, 2, 3 | Q | Q | Q |
| G228S^a^ | 1, 2 | S | L | L |
| T318I^a^ | 1, 2 | I | I | I |
| H103Y^b^ | 2, 4, 5 | S | N | N |
| N154D^b^ | 1, 2, 4 | C | C | C |
| T156A^b^ | 2, 4, 5 | Y | Y | Y |
| N182K^b^ | 2, 4 | S | S | S |
| D187G^b^ | 2, 6 | N | N | N |
| E190G^b^ | 2, 6 | D | D | D |
| Q196R^b^ | 2, 6 | G | G | G |
| N220K^b^ | 1, 2 | G | G | G |
| Q222L^b^ | 1, 2, 4, 5 | S | S | S |
| G224S^b^ | 1, 5, 7 | L | L | L |
| E225K^b^ | 2, 6 | N | N | N |
| S227N^b^ | 2, 6 | R | R | R |
| E255K^b^ | 1, 2, 6 | N | N | N |
| T314I^b^ | 2, 5 | H | H | H |

^a^H5 numbering.

^b^H3 numbering.

^c^Reference:

1. Imai M, Watanabe T, Hatta M, Das SC, Ozawa M, Shinya K, Zhong G, Hanson A, Katsura H, Watanabe S, Li C, Kawakami E, Yamada S, Kiso M, Suzuki Y, Maher EA, Neumann G, Kawaoka Y: **Experimental adaptation of an influenza H5 HA confers respiratory droplet transmission to a reassortant H5 HA/H1N1 virus in ferrets.** *Nature* 2012, **486**(7403)**:**420-428.

2. Xiong X, Coombs PJ, Martin SR, LiuJ, XiaoH, McCauley JW, Locher K, Walker PA, Collins PJ, Kawaoka Y, Skehel JJ, Gamblin SJ: **Receptor binding by a ferret-transmissible H5 avian influenza virus.** *Nature* 2013, **497**(7449)**:**392-396.

3. Ha Y, Stevens DJ, Skehel JJ, Wiley DC: **X-ray structures of H5 avian and H9 swine influenza virus hemagglutinins bound to avian and human receptor analogs.** *Proc Natl Acad Sci USA* 2001, **98**(20)**:**11181-11186.

4. Herfst S, Schrauwen EJ, Linster M, Chutinimitkul S, de Wit E, Munster VJ, Sorrell EM, Bestebroer TM, Burke DF, Smith DJ, Rimmelzwaan GF, Osterhaus AD, Fouchier RA: **Airborne transmission of influenza A/H5N1 virus between ferrets.** *Science* 2012, **336**(6088)**:**1534-1541.

5. Neumann G, Macken CA, Karasin AI, Fouchier RAM, Kawaoka Y: **Egyptian H5N1 Influenza Viruses – Cause for Concern?** *PLoS Pathogens* 2012, **8**(11)**:**e1002932.

6. Chen LM, Blixt O, Stevens J, Lipatov AS, Davis CT, Collins BE, Cox NJ, Paulson JC, Donis RO: **In vitro evolution of H5N1 avian Influenza virus toward human-type receptor specificity.** *Virology* 2012, **422**(1)**:**105-113.

7. Yamada S, Hatta M, Staker BL, Watanabe S, Imai M, Shinya K, Sakai-Tagawa Y, Ito M, Ozawa M, Watanabe T, Sakabe S, Li C, Kim JH, Myler PJ, Phan I, Raymond A, Smith E, Stacy R, Nidom CA, Lank SM, Wiseman RW, Bimber BN, O'Connor DH, Neumann G, Stewart LJ, Kawaoka Y: **Biological and structural characterization of a host-adapting amino acid in influenza virus.** *PLoS Pathog* 2010, **6**(8)**:**e1001034.
